# Molecular Pathology, Diagnostics, and Therapeutics of Nephropathy

**DOI:** 10.3390/ijms232416006

**Published:** 2022-12-16

**Authors:** Andreas Kronbichler, Vladimir Tesar

**Affiliations:** 1 Department of Medicine, University of Cambridge, Trinity Ln, Cambridge CB2 1TN, UK; 2 Cambridge University Hospitals, Addenbrooke’s Hospital, Hills Road, Cambridge CB2 0QQ, UK; 3 Department of Nephrology, First Faculty of Medicine, General University Hospital, Charles University, U Nemocnice 2, 128 08 Prague, Czech Republic

Years of standing still have ended, and the field of nephrology has seen a plethora of clinical trials, changing the therapeutic landscape of chronic kidney disease (CKD) and immune-mediated kidney disease management. Such progress is paralleled by investment in the refinement of diagnostic approaches; for example, molecular pathology, a means to decipher the pathways involved in kidney diseases. For such purposes, researchers use human tissue specimens (kidney biopsy samples), blood, and urine and perform experiments using mouse models or cell cultures, such as proximal tubular cells. Kidney biopsies remain valuable to a final diagnosis and have achieved enormous value in predicting prognosis in several kidney diseases, such as anti-neutrophil cytoplasmic antibody (ANCA)-glomerulonephritis [1,2]. Likewise, repeat kidney biopsies to assess renal-limited disease activity have become a reality in lupus nephritis (LN) [3]. This Special Issue includes 13 articles, of which eight were original articles and warrant further discussion. They dealt with the whole width of renal research, ranging from studying hypoxia in cell culture to a meta-analysis of the transcriptome changes in peritoneal dialysis (PD).

Bernardo-Bermejo et al. studied the metabolic changes in the human proximal tubular cell line HK-2 under normal conditions and when hypoxia was induced. Under hypoxic conditions, hypoxia-inducible factor (HIF)-1-alpha starts to increase at 5 h, and the authors asked if metabolic changes are indeed observed at an earlier stage and used two time points for their initial analysis at 0.5 and 5 h. Further experiments were carried out at a later stage, namely 24 and 48 h. More significant changes were observed in the extracellular compared to the intracellular fluid, and in general, the metabolic re-wiring of cells increases over time when hypoxic conditions are ongoing but started as early as 0.5 h. The authors identified a total of 16 and 141 statistically significant features [4] and argue that some of these factors are associated with either changes observed in acute kidney injury (AKI) or CKD.

Casili et al. studied the effects of KYP2047, a selective inhibitor of prolyl oligopeptidase (POP), in an in vivo model of kidney ischemia and reperfusion. The treatment with KYP2047 at higher doses (1 and 5 mg/kg, intraperitoneal administration) reduced the histologic markers of kidney injury (such as tubular atrophy (TA) and interstitial fibrosis (IF)), negatively modulated inflammation through the NF-кB pathway, and thereby restored kidney function. In addition, apoptosis was reduced, and treatment with KYP2047 reduced the markers of angiogenesis and fibrosis, such as vascular endothelial growth factor (VEGF) and transforming growth factor (TGF)-ß1 [5]. POP inhibition might be carried forward to develop therapies that aim to reduce the damage to kidneys caused by ischemia and reperfusion injury, such as after kidney transplantation.

The impact of TGF-ß1 on fibrosis in different renal segments is well known. Hwang et al. studied fibrosis-mimicking models using 3-dimensional (3D) co-culture devices designed with layers of tubule interstitium, including epithelial, fibroblastic, and endothelial layers. In the next step, HK-2 cells, human umbilical-vein endothelial cells (HUVECs), and patient-derived renal fibroblasts were introduced, and the effects of TGF-ß and TGF-ß inhibitors on this model were studied in detail. In the 3D model, treatment with TGF-ß1 significantly increased the formation of thick lines in endothelial cells; these changes were partially reversed by the TGF-ß1 inhibitor treatment. The authors further found a higher expression level of interleukin (IL)-1ß, fibroblast growth factor (FGF)-2, TGF-ß2 and TGF-ß3 in the 3D model, while the expression of TGF-ß1 was similar in comparison to the 2D model [6]. In conclusion, this newly established 3D model might be suitable for studying fibrosis in renal segments, but further confirmatory studies are required.

Sörensen-Zender et al. also focused on kidney fibrosis and strategies to mitigate fibrotic changes. For this purpose, they used C57Bl/6J mice and treated the mice with Zinc-alpha2-glycoprotein (AZGP1) and, in a further step, generated a transgenic mouse strain with the overexpression of AZGP1. In the first set of experiments, mice underwent unilateral ureteric obstruction (UUO), and the kidneys were examined after fourteen days. Therapy with recombinant AZGP1 led to the better preservation of tubular integrity, reduced the deposition of collagen, and reduced the expression of fibrosis and kidney injury markers, as indicated in part by a lower expression of *Havcr1*, coding for kidney injury marker-1, *Acta2*, coding for alpha-smooth muscle actin, and *Col1a1*, coding for collagen type 1. A similar, yet less extensive, reduction in the hallmarks of fibrotic kidney disease was observed in transgenic AZGP1-overexpressing mice [7]. This study further underlines that AZGP1 might be a relevant counterpart to TGF-ß1, and treatment with AZGP1 might reduce fibrotic changes in the kidney.

Frydlova et al. analyzed the urine samples of patients with ANCA-associated vasculitis, an area of particular interest to study changes in kidney function. Next-generation sequencing (NGS) of urinary extracellular vesicles (uEV) was performed to identify uniquely regulated microRNAs (miRNAs), followed by a single-target real-time PCR to confirm the results. For this purpose, 10 and 24 patients were recruited and compared to unmatched healthy controls. NGS identified 161 and 238 differentially expressed miRNAs by using DESeq2 and edgeR, and as expected, the uEV-derived miRNA transcripts differed between the groups. In the confirmation phase, nine selected miRNAs were evaluated in 24 patient samples. Using this approach, some of the miRNAs were differentially regulated in comparison to the NGS experiments, i.e., miR-26a-5p, while four of the others were confirmed by the real-time PCR experiments [8]. From these results, it seems difficult to draw conclusions on the pathogenesis of ANCA-associated vasculitis, one of the aims of the study. Nonetheless, enrichment analysis identified several pathways relevant to the etiopathogenesis of ANCA-associated vasculitis, including the VEGF and VEGFR signaling network and the thrombin/protease-activated receptor (PAR) pathway [8].

Autosomal dominant polycystic kidney disease (ADPKD) has an uncertain clinical course, ranging from only a modest decline in estimated glomerular filtration rate (eGFR) over time to progressive decline and end-stage kidney disease within a couple of years. Leierer et al. asked whether a subset of eight biomarkers is differentially expressed in the urine and blood of patients with ADPKD (n = 37). These biomarkers were obtained from studying the reported molecular features of ADPKD and included angiotensinogen (AGT), apelin (APLN), arginine vasopressin (AVP), epidermal growth factor (EGF), TGF-ß1, tumor necrosis factor (TNF), VEGF-A, and vimentin (VIM). The expression levels were compared to an eGFR-matched CKD population and healthy controls. In brief, most biomarkers showed no difference between ADPKD and CKD, while the expression of some was lower compared to healthy individuals (i.e., urinary EGF), others were higher (i.e., serum VIM). Plasma AVP, nonetheless, was higher in patients with ADPKD in comparison to CKD, which can be explained by the underlying pathophysiology [9]. Together, the expression of these biomarkers rather reflects CKD progression than informing us about specific changes observed in ADPKD.

Sodium homeostasis and blood pressure are tightly regulated by the renin–angiotensin system (RAS). The authors hypothesized that the knock-out of *Ercc1*, which is pivotal in DNA repair pathways and accelerates aging when mutated, would increase the tempo of kidney failure in mice. For this purpose, progeroid *Ercc1^d/-^* mice were created, and the impact of the knock-out was confirmed using kidney histology at 24 weeks, indicating hallmarks of chronic kidney damage such as glomerulosclerosis. In contrast to the hypothesis, the plasma renin levels of these animals were lower, while the intrarenal renin activity was increased in comparison to wild-type mice. This study provided evidence that RAS activity, as measured in the kidney and in the plasma, does not necessarily run in parallel. However, the probe ReninSense^®^ might be a useful and non-invasive tool to measure intrarenal renin activity [10], but this warrants further study.

Evgeniou et al. analyzed twelve transcriptome datasets of PD patients using different samples (PBMCs, mesothelial cells, peritoneal cells, and omental arterioles). A total of 3179 differentially expressed transcripts were found that mapped to 2591 unique differentially expressed genes (DEGs). The analysis of mesothelial cells provided the largest set of DEGs, with a total number of 2286. Gene ontology (GO) identified 41 enriched biological processes, of which angiogenesis was the most prominent one, followed by cell adhesion, cell division, and cell migration. The authors further focused on receptor-receptor complexes and receptor-ligand interactions and found that six and 70 were uniquely dysregulated, respectively. In the latter group, extracellular matrix (ECM) organization driven by integrin subunit beta 1 (ITGB1) and ITGB2 was most prominent [11]. This study highlights that angiogenesis and mechanisms involved in ECM re-organization play a pivotal role in patients undergoing chronic PD treatment.

In conclusion, this Special Issue provided relevant insights into modern techniques to study kidney disease, especially fibrotic kidney disease, ADPKD, ANCA-associated vasculitis, and patients undergoing chronic PD. Overall, the provided results will further help to increase our understanding of kidney disease in more detail and will eventually lead to individualized treatment approaches for the plethora of kidney diseases.
